# The use of artificial neural networks in electrostatic force microscopy

**DOI:** 10.1186/1556-276X-7-250

**Published:** 2012-05-15

**Authors:** Elena Castellano-Hernández, Francisco B Rodríguez, Eduardo Serrano, Pablo Varona, Gomez Monivas Sacha

**Affiliations:** 1 Grupo de Neurocomputación Biológica, Departamento de Ingeniería Informática, Escuela Politécnica Superior, Universidad Autónoma de Madrid, Campus de Cantoblanco, Madrid 28049, Spain

**Keywords:** Electrostatic force microscopy, Thin films, Artificial neural networks

## Abstract

The use of electrostatic force microscopy (EFM) to characterize and manipulate surfaces at the nanoscale usually faces the problem of dealing with systems where several parameters are not known. Artificial neural networks (ANNs) have demonstrated to be a very useful tool to tackle this type of problems. Here, we show that the use of ANNs allows us to quantitatively estimate magnitudes such as the dielectric constant of thin films. To improve thin film dielectric constant estimations in EFM, we first increase the accuracy of numerical simulations by replacing the standard minimization technique by a method based on ANN learning algorithms. Second, we use the improved numerical results to build a complete training set for a new ANN. The results obtained by the ANN suggest that accurate values for the thin film dielectric constant can only be estimated if the thin film thickness and sample dielectric constant are known.

**PACS:** 07.79.Lh; 07.05.Mh; 61.46.Fg.

## Background

When electrostatic force microscopy (EFM) [1-6] is working at the nanoscale, several interacting parameters have a strong influence in the signal [7]. Since the electrostatic force is a long-range interaction, macroscopic parameters such as the shape of the tip or the sample thickness can strongly modify the electrostatic interaction [8,9]. However, in many experimental situations, it is not possible to obtain accurate values for all of these parameters, and it is very difficult to achieve quantitative experimental results [10]. Previous results [11] have shown that artificial neural networks (ANNs) [12] are a useful tool to characterize dielectric samples in highly undetermined EFM systems. Using known force vs. distance curves as inputs for their training, ANNs have been able to estimate the dielectric constant of a semi-infinite sample in a system where the tip radius and shape were not known.

In this paper, we demonstrate that ANNs can be used to improve the accuracy of numerical simulations in EFM and to quantitatively estimate the thin film dielectric constant from vertical force curves. First, we compare standard minimization and ANN techniques, demonstrating that ANN techniques provide a better control of the final result of the simulation. The improved numerical results are also used to create a complete training set of an ANN that estimates the dielectric constant of a thin film placed over a dielectric sample.

As it has been shown before [11], ANNs are able to estimate physical magnitudes in highly undetermined systems. In this article, we train an ANN with a complete thin film sample to study the necessity of knowing the geometry of the sample in the estimations of the thin film dielectric constant. Although the influence of the thin film thickness is much larger than that of the substrate dielectric constant, we demonstrate that accurate values of the thin film dielectric constant can only be obtained when both magnitudes are known.

## Methods

### Artificial neural network formalism for the calculation of electric fields

**Figure 1:**
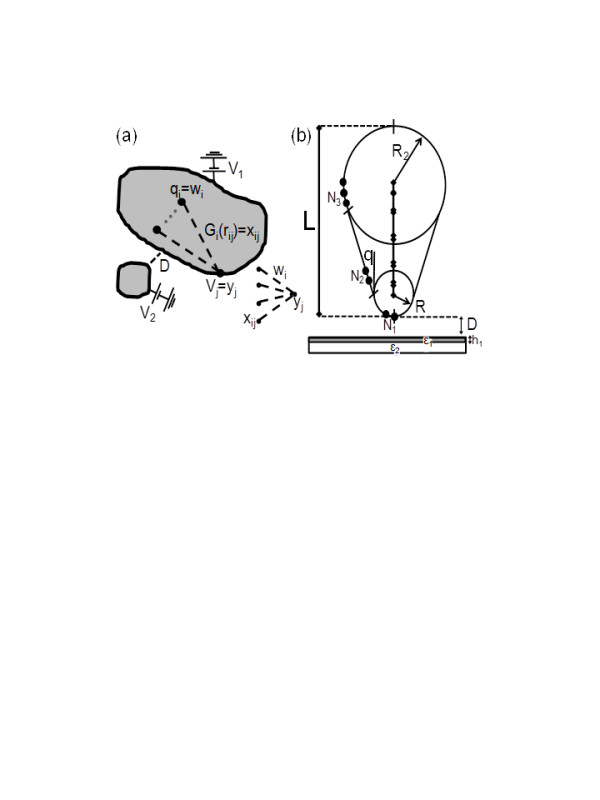
**Two metallic objects with different applied voltages and electrostatic force microscope tip and sample.** (**a**) Schematic representation of a system of two metallic objects with different applied voltages *V*_1_ and *V*_2_. Its equivalent ANN is also shown as an inset. (**b**) Scheme of an electrostatic force microscope tip and sample. The tip surface has been divided in three regions with a finite number of points (N_1_, N_2_, and N_3_). The tip and sample are characterized by eight parameters: the tip sample distance *D*, the tip apex radius *R*, the tip length *L*, the cone half-angle *θ*, the radius *R*_2_, the thin film thickness *h*_1_, the thin film dielectric constant *ϵ*_1_, and the sample dielectric constant *ϵ*_2_.

To briefly illustrate how ANNs can be related to the problem of estimating unknown parameters in EFM setups, let us focus in the scheme shown in Figure 1a. Here, we have a set of metallic objects that are connected to a battery that provides a constant electric potential. The calculation of electric magnitudes such as the electrostatic potential or the force between these elements is, in general, very difficult, and only a few specific geometries can be analytically calculated [13]. To solve electrostatic problems with arbitrary geometries, an algorithm called the generalized image charge method [14,15] (GICM) has been developed. The GICM replaces the surface charge density by a set of charges inside the metallic objects. The value, position, and number of charges are obtained after a standard least-squares minimization (LSM) routine for the electrostatic potential at the metallic surfaces. An alternative to the LSM is to use the ANN formalism by considering the value of the charges *q*_i_ as the weights *w*_i_ and the potential at the metallic surfaces *V*_j_ as the expected output values *y*_j_ (see inset of Figure 1a). The input patterns *x*_ij_ play the role of the Green functions *G*_i_(*r*_ij_*σ*_i_), where *r*_ij_ is the distance between the i-charge element and the j-surface point. *σ*_i_ represents the geometrical parameters that may be used to adequately calculate the electrostatic potential generated by the i-charge element (for example, if the element is a charged line, *σ*_i_ would represent the length of the line). Following this formalism, the electrostatic potential *V*_j_ can be expressed as

(1) $$V_{j}=\sum_{i=1}^{N_{\text{C}}}q_{\text{i}}G_{\text{i}}(r_{\text{ij}},\sigma_{\text{i}})=\sum_{i=1}^{N_{\text{C}}}w_{\text{i}}x_{\text{ij}}\text{,}$$

where *N*_C_ is the number of charged elements *q*_i_ inside the tip. The most right-hand-side term in Equation 1 represents the electrostatic potential in the notation of a single-output ANN, where *x*_ij_ represents the inputs to the output neuron *y*_j_, and *w*_i_ are the connection weights from the inputs (*i* = 1,…,) to this neuron (see Figure 1a). A neural network learns by example. The task of the learning algorithm of the network (i.e., the delta rule, backpropagation, etc. [12,16]) is to determine *w*_i_ (i.e., *q*_i_) from available *x*_ij_ data. Previous knowledge can help us to decide the best values for *N*_C_ (by the selection of the number of neurons) and *G*_i_ (by the selection of input patterns).

**Figure 2:**
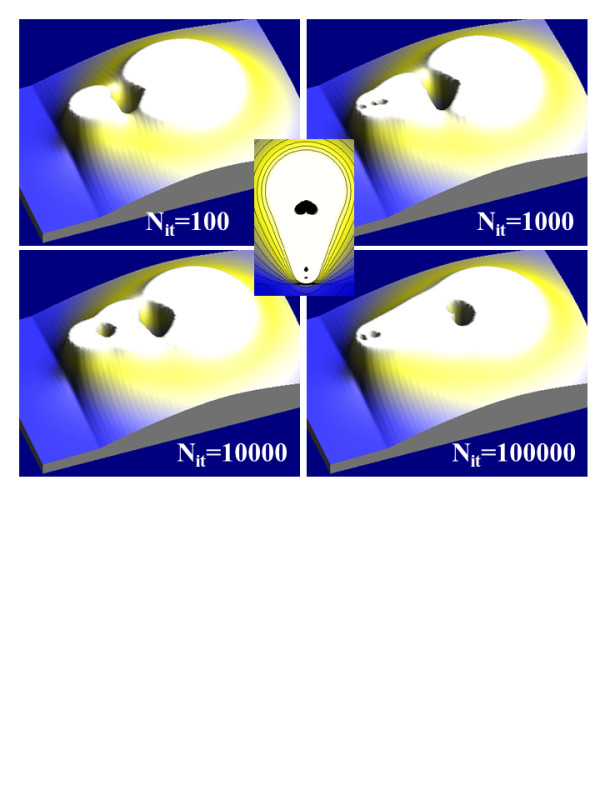
P**otential distribution around an EFM tip.** 3D representation of the potential distribution around an EFM tip (*L* = 10 *R*, *θ* = 20°) over a dielectric sample (*D* = 0.1 *R*, *ϵ* = 10) as a function of the ANN iterations in the learning process *N*_it_. 2D equipotential distribution in the middle of the image corresponds to the results obtained by the standard LSM.

To compare both minimization techniques (LSM and ANN), we have simulated the EFM shown in Figure 1b. To illustrate the advantages of using the ANN minimization routine, we have calculated the *q*_i_ coefficients for the tip-sample distance described in Figure 1b with both the ANN and standard LSM routines. We have used the winGICM software v1.1 [17] which also uses ANNs to estimate the number of punctual charges and the number of segments (4 and 12, respectively). The LSM lowest error (located at *x* = 0.9876 *Rz* = 0.8896 *R*) at the tip surface was 0.0019 *V*_0_, where *V*_0_ is the voltage applied to the tip. In Figure 2, we show the electrostatic potential distribution obtained for different numbers of iterations *N*_it_ in the training process. We initialized *q*_i_ = 0 and fixed the learning rate to 0.1. When *N*_it_ = 100,000, the equipotential distribution looks identical than that obtained by the LSM. However, the highest error at the tip surface is 0.0076 *V*_0_ (four times larger than that from LSM). At this point, it seems that LSM is a better minimization technique since it gives a lower error and does not use any iterative process. However, the *q*_i_ values obtained by the LSM are not adequate for several physical applications. As we can see in Table1, the ANN *q*_i_ absolute values are much smaller than those from LSM. This fact is not important when *q*_i_ do not have any physical meaning. However, in our case, *q*_i_ correspond to the charge inside the tip and must be used to calculate electric magnitudes like the capacitance or the electrostatic force *F* (used in the following section). By using ANN *q*_i_ values, these magnitudes can be calculated with improved accuracy since the low values of the charges strongly reduce the numerical noise. In conclusion, the ANN minimization allows the user to choose the balance between numerical accuracy and physical meaning of the simulations to adapt them to the necessity of the problem.

**Table 1:** Coefficients obtained by the ANN and LSM algorithms for an EFM system

**Coefficients**	**ANN**	**LSM**
*q*_i_		
q1	−0.26	417.40
q2	1.75	−2.31
q3	−1.54	2.43
q4	0.98	−0.82
*L*_i_		
L1	−0.032	−12,582.71
L2	−0.15	2,194.47
L3	−0.30	−157.42
L4	0.90	5.51
L5	0.41	0.015
L6	0.13	0.48
L7	1.32	−0.39
L8	−7.31	26.26
L9	10.16	−445.25
L10	11.51	3,126.11
L11	7.75	−9,298.52
L12	4.42	9,311.48

*L* = 10 *R*, *θ* = 20°, *D* = 0.1 *R*, and *ϵ* = 10. *q*_i_ and *L*_i_ correspond to punctual charges and charged segments inside the tip, respectively.

## Results and discussion

### Thin film dielectric constant estimation

**Figure 3:**
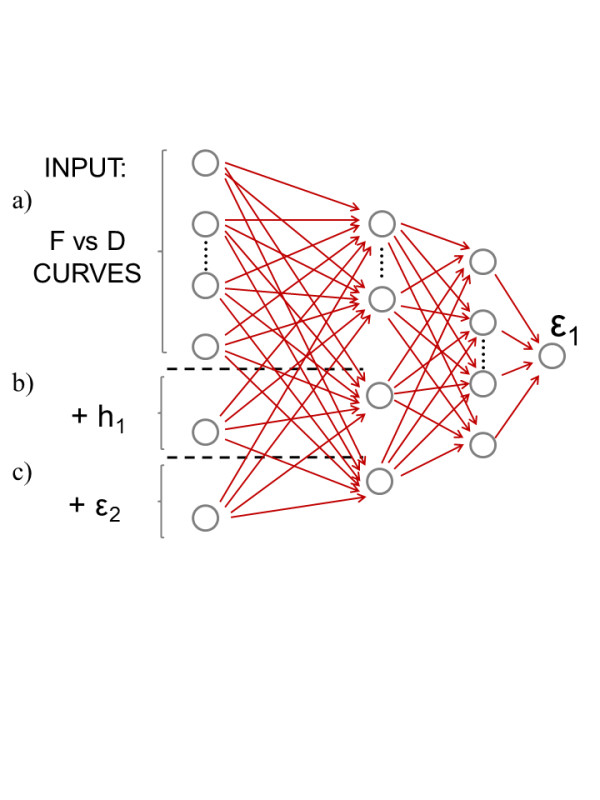
**Scheme of the ANN used to estimate*ϵ***_**1**_**.** Inputs are (**a**) *F* vs. *D* curves; (**b**) *F* vs. *D* curves and *h*_1_ thickness; and (**c**) *F* vs. *D* curves, *h*_1_ thickness, and *ϵ*_2_.

**Figure 4:**
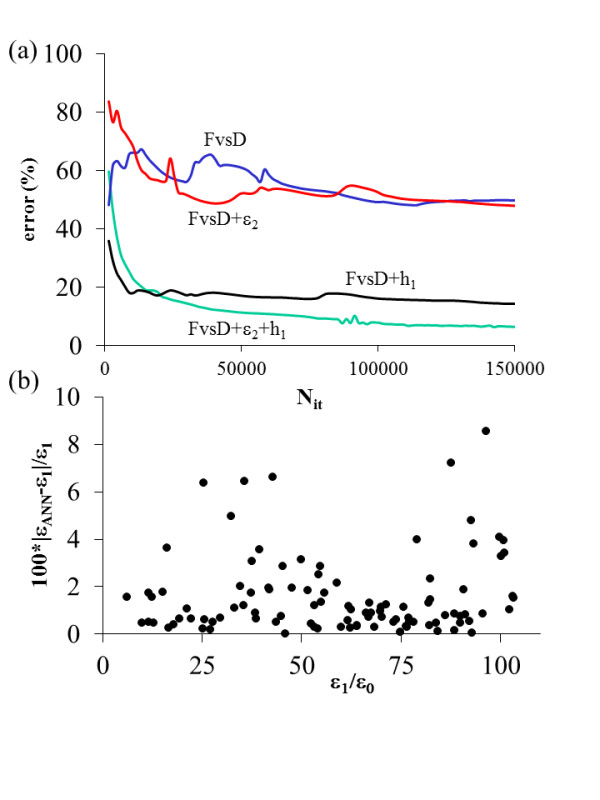
**Test error vs. number of iterations and ANN estimation.** (**a**) Test error vs. number of iterations in the ANN learning process for different ANN inputs. (**b**) ANN estimation of the thin film dielectric constant for the test set for the case where *F* vs. *D*, *ϵ*_2_, and *h*_1_ are included as inputs.

In this section, we are going to use the GICM force vs. tip-sample distance (*F* vs. *D*) curves for an EFM tip over a thin film to train an ANN that will be able to estimate the dielectric constant *ϵ*_1_ of the thin film with thickness *h*_1_ (see Figure 1b). The thin film will be placed over a semi-infinite dielectric substrate characterized by its dielectric constant *ϵ*_2_. It is worth noting that in realistic systems where *h*_1_ is very small, *ϵ*_1_ should be considered an effective [6] dielectric constant since several nanoscale effects can modify the response of the thin material and change the *ϵ*_1_ value. Some examples of physical phenomena that could affect *ϵ*_1_ are the roughness of the thin film surface, the presence of a water layer over the thin film [5], or the finite amount of free charge due to the small size of the film. The ANN architecture is shown in Figure 3. We used a multilayer perceptron with sigmoid activation functions. The input layer is composed of 20 neurons for the *F* vs. *D* curves that are calculated for *D* = {2.5, 5,…,50} nm. Additional neurons are added in the cases where *ϵ*_2_ and *h*_1_ are included as input values. We used two hidden layers composed of 10 neurons with no bias applied. The output layer contains a single neuron which provides the estimate values for *ϵ*_1_. We have considered three different inputs: the *F* vs. *D* curves, *h*_1_, and *ϵ*_2_. GICM *F* vs. *D* curves included in the training were calculated for *ϵ*_1_ = {5, 15, 25,…,105}, *ϵ*_2_ = {5, 15, 25,…,105}, and *h*_1_ = {1, 2,…,10}. The ANN has been tested with 100 *F* vs. *D* curves (not used during the training) with randomly selected *ϵ*_1_*ϵ*_2_ (between 5 and 105), and *h*_1_ (between 1 and 10) values. As we can see in Figure 4a, although the ANN is able to estimate *ϵ*_1_ when *F* vs. *D* and *h*_1_ (excluding *ϵ*_2_) are used as input parameters, it gives the best results when all the input parameters are included. In Figure 4b, we show the error obtained by the ANN when all the inputs are included. The error is always smaller than 9% for all the *ϵ*_1_ values.

The ANN can be used with realistic experimental curves without any previous treatment, which is one of the advantages of using this technique [11]. In this case, experimental curves with a high error could make the ANN give wrong *ϵ*_1_ estimations. This problem can be easily solved by training the ANN with a mixture of experimental and numerical *F* vs. *D* curves. This strategy would make the ANN more robust against experimental noise (by the use of experimental curves) and still effective on the *ϵ*_1_ estimations (by the use of a whole set of numerical curves).

Recently, a simple analytical expression has been developed that demonstrates that a sample composed by a thin film over a dielectric substrate gives the same response as that of a semi-infinite uniform dielectric sample [18]. The fact that different combinations of *ϵ*_1_*ϵ*_2_, and *h*_1_ can correspond to the same effective dielectric constant is in agreement with the results found in Figure 4a since including *ϵ*_2_ and *h*_1_ as input values improves the ANN performance in the *ϵ*_1_ estimations.

## Conclusions

We have demonstrated that ANNs can strongly improve the efficiency of the characterization of samples by electrostatic force microscopy. First, we have demonstrated that the generalized image charge method can be modified to use a neural network minimization algorithm. Using this technique, we have increased the accuracy of the electrostatic force and capacitance calculations. By using electrostatic force simulations, we have been able to train an ANN to estimate the dielectric constant of thin films. The analysis of the results of the ANN suggests that the thin film dielectric constant can only be obtained when the thin film thickness and the dielectric nature of the sample are known. Note that the methods explained in this paper can be easily applied to experimental data by providing this kind of input to the ANN. If enough data are available, experimental curves can be used for the ANN training alone or together with theoretical curves.

## Abbreviations

ANNs, Artificial neural networks; EFM, Electrostatic force microscopy; F vs. D, Force vs. tip-sample distance; GICM, Generalized image charge method; LSM, Least-squares minimization.

## Competing interests

The authors declare that they have no competing interests.

## Authors' contributions

ECH carried out the numerical simulations. FBR and ES participated in the design of the artificial neural networks and mathematical formalism. PV participated in the design of the artificial neural networks and helped draft the manuscript. GMS conceived the study, participated in its design and coordination, and drafted the manuscript. All authors read and approved the final manuscript.
